# Mass-production of human dendritic cells in accordance with gmp for clinical studies

**DOI:** 10.1186/2051-1426-3-S2-P207

**Published:** 2015-11-04

**Authors:** Yui Harada, Noriko Yasuda, Yoshikazu Yonemitsu

**Affiliations:** 1 R&D Laboratory for innovative Biotherapeutics, Graduate School of Pharmaceutical Sciences, Fukuoka, Japan; 2 R&D Laboratory for Innovative Biotherapeutics, Graduate School of Pharmaceutical Sciences, Kyushu University, Fukuoka, Japan

## Background

Although clinical studies have established that antigen-loaded dendritic cells (DCs) vaccines are safe and promising therapy for various kinds of malignancies, their clinical efficacy remains to be established. Issues that limit the clinical efficacy of DC-based immunotherapy, as well as the difficulty of the industrial production of DCs, are largely due to the limited number of autologous DCs available from each patient (Kato T., et al. *Neoplasia* 2010), and it is necessary to prepare an enough number of DCs for effective treatments of tumors. In this study, therefore, we attempted to expand functional human DCs of cancer patients *ex vivo*.

## Methods

The method to produce DCs, prepared in accordance with Good Manufacturing Practice (GMP) guidelines, is an optimization of *ex vivo* preparation method for generating large numbers of DCs from peripheral blood mononuclear cells (PBMCs) obtained by leukapheresis for clinical studies. Several fraction-depleted PBMCs were expanded and differentiated into DCs in the presence of optimal cytokine cocktails for 3-4 weeks by floating cultivation.

## Results

By this method, about 1x10^9^ CD11c^+^ cells could be obtained. These cells had typical features (endocytotic activity, expression of HLA-DR, adhesion molecules, chemokine receptor and co-stimulatory molecules, production of inflammatory cytokines, allo-MLR activity and positive for tetramer assay) of conventional myeloid DCs *in vitro*. Therefore, the concept of DC expansion should contribute significantly to the progress of DC immunotherapy.

## Conclusions

We established a new culture method to expand human DCs. Expanded DCs had properties that were required to obtain therapeutic gain. Thus, we expect that this technology will improve therapeutic gain of cancer and alleviate patients' burden of apheresis, and be able to contribute largely to both basic and clinical research of human cancer immunotherapy.

